# Can the mucosal attacks of Behçet’s disease be predicted?

**DOI:** 10.1007/s00403-023-02805-0

**Published:** 2024-01-18

**Authors:** Selda Pelin Kartal, Gamze Taş-Aygar, Ümmü Gül Erdem, Ali Yalçındağ, Müzeyyen Gönül

**Affiliations:** 1 University of Health Sciences, Etlik City Hospital, Dermatology Clinic, Ankara, 06170 Turkey; 2 University of Health Sciences, Etlik City Hospital, Microbiology Clinic, Ankara, 06170 Turkey; 3 Universty of Health Sciences, Dışkapı Yıldırım Beyazıt Training and Research Hospital, Biochemistry Clinic, Ankara, 06110 Turkey; 4 University of Health Sciences Etlik City Hospital, Dermatology Clinic, Ankara, 06170 Turkey

**Keywords:** Behçet’s disease, Procalcitonin, Oral aphthous ulcer, Mucosal involvement

## Abstract

The primary objective of this study was to investigate the association between disease activity and serum and salivary procalcitonin (Pct) levels in patients with Behçet’s disease (BD). The study included patients diagnosed with BD and age-matched healthy volunteers (*N*: 48, *N*: 32). Serum and salivary Pct levels were quantified using enzyme-linked immunosorbent assay (ELISA) in the laboratories of Diskapi Yildirim Beyazit Training and Research Hospital. No significant disparity was observed in serum and salivary Pct levels between the patient and control groups (*p* > 0.05). Furthermore, there was no noteworthy correlation between disease activity and serum and salivary Pct values (*p* > 0.05). However, the serum Pct level in patients with active oral ulcers was significantly elevated compared to those without active oral ulcers (*p*: 0.003). Serum Pct emerges as a valuable marker for monitoring oral aphthous ulcer attacks within the patient population.

## Introduction

Behçet’s disease (BD), initially delineated in 1937 by the Turkish dermatologist Professor Dr. Hulusi Behçet, manifests with recurrent oral and genital aphthous ulcers and hypopionic uveitis. This multisystem vasculitis impacts individuals in their young adulthood, affecting both men and women. The condition significantly diminishes the quality of life and carries the potential for serious disability and premature death 1, 2.

In 80% of patients, oral aphthous ulcers represent the initial manifestation of BD. Consequently, there is a prevailing hypothesis suggesting that oral environmental factors may exert a substantial influence on the etiology and pathogenesis of BD. Studies have indicated a heightened rate of colonization of oral mucosa streptococci in individuals with BD compared to those with recurrent oral aphthous syndrome and the general population 3.

Procalcitonin (Pct), a 116 amino acid peptide, exhibits elevated levels in streptococcal infections as opposed to infections caused by other microorganisms 4. Pct, a 116 amino acid peptide, exhibits elevated levels in streptococcal infections as opposed to infections caused by other microorganisms. The association between oral streptococcal colonization in Behçet’s disease (BD) pathogenesis and the heightened Pct levels in streptococcal diseases in contrast to other bacterial infections has led to the proposition that Pct may serve as a valuable marker for evaluating disease activity. In consideration of the disease’s pathogenesis, this study explores the correlation between disease activity and serum, as well as salivary, Pct levels in patients diagnosed with BD.

## Material and methods

The study protocol obtained approval from the local institutional ethics committee and adhered to the International Ethical Guidelines as outlined in the Declaration of Helsinki. Inclusion criteria comprised patients aged between 18 and 70, under the follow-up care for BD, and diagnosed according to the criteria of the International Behçet Study Group at the chronic diseases outpatient clinic of the Dermatology Clinic of Health Sciences University Ankara Dışkapı Yıldırım Beyazıt Training and Research Hospital. The inclusion of patients in the study spanned those who applied to our clinic between June 2017 and January 2017 and met the specified criteria.

In the selection of the control group, careful consideration was given to include the minimum number of healthy volunteers necessary to preserve the study’s reliability. Patients with a diagnosis of BD and healthy volunteers within the same age group were apprised of the study details, and signed consents were obtained from those who expressed willingness to participate. Subsequently, both patients and healthy volunteers were incorporated into the study. Data including age, sex, principal types of BD involvement (such as oral and genital ulcerations, and manifestations in the cutaneous, gastrointestinal, articular, ocular, neurologic, and vascular domains), and treatment details were systematically recorded. The assessment of BD activity in patients was conducted utilizing the Behçet’s Disease Current Activity Form, a tool developed by the International Society for Behçet Disease. The data obtained through this form served to delineate the activity levels of BD. Notably, individuals diagnosed with concomitant medical conditions such as diabetes, hypertension, ischemic heart disease, psoriasis, inflammatory bowel disease, and rheumatoid arthritis were excluded from the study. Additionally, exclusion criteria encompassed patients with severe infective conditions (sepsis, meningitis, infective endocarditis), those undergoing massive blood transfusion, individuals experiencing liver and kidney failure, as well as those with drug and alcohol dependence, depressive disorders, psychiatric diseases, and multiple organ failure. Furthermore, patients with compromised oral hygiene, dental abscess, tooth/gum infections, and those receiving local or systemic antibiotic treatment in the oral cavity were also excluded from the study. The control group comprised healthy volunteers who underwent routine physical examinations at our hospital. The patient and control groups were matched with respect to age and gender. Individuals with other autoimmune diseases, liver or kidney disease, hematologic disorders, diabetes, cancer, or acute or chronic infections were excluded from the study. Following an 8-h fasting period, non-stimulated saliva and blood samples were collected, then centrifuged at 3000 rpm for 10 min at 4 °C. The plasma was subsequently stored at − 80 °C in the biochemistry department, while saliva samples were stored at – 20 °C in the microbiology department until analysis. Serum and salivary Pct levels were measured by enzyme-linked immunosorbent assay (ELISA) using commercial kits (Sandwich-ELISA, Eastbiopharm, CK-E90535, Hangzhou, Zhejiang, China) in Diskapi Yildirim Beyazit Training and Research Hospital Laboratories. Pct concentration of the samples was interpolated from the standard curve. The detection range for the assay was 5–2000 pg/ml for Pct.

The data obtained were transferred to the computer environment and evaluated with the SPSS (v.15.0) statistics package program. The compliance of the data to normal distribution was evaluated with the Kolmogrov–Smirnov test. Analysis of data inappropriate to normal distribution was conducted using Mann–Whitney *U* and Spearman Correlation test; analysis of data appropriate to normal distribution was conducted using Student’s *t* test. Chi-square analysis was employed to assess categorical variables in the study.

## Results

The study group comprised 48 subjects diagnosed with BD, consisting of 11 males and 37 females, with a mean age of 44.63 years (age range 18–70 years). A control group of 32 healthy volunteers was included, comprising 9 males and 25 females, with a mean age of 42.06 years (age range 21–62 years).

The prevalence of active clinical manifestations within the patient group was as follows: 32 individuals with oral ulceration (66.7%), 4 with genital ulceration (8.3%), 4 with ocular involvement (8.3%), 6 with erythema nodosum (EN) (12.5%), 12 with papulopustular eruption (PPE) (25%), 9 with arthritis (18.8%), and 3 with neurological involvement (6.3%). Notably, there were no patients exhibiting vascular involvement (Table 1).

**Table 1:** Laboratory and clinical findings of patient and control groups

	Patient	Control	
Sex	11 M, 37 F	9 M, 25 F	
Age	44,63	42,06	
Oral ulceration	32 (66.7%)	–	
Genital ulceration	4 (8.3%)	–	
Ocular involvement	4 (8.3%)	–	
Erytema nodosum	6 (12.5%)	–	
Papulopustular eruption	12 (25%)	–	
Arthritis	9 (18.8%)	–	
Neurological involvement	3 (6.3%)	–	
Vascular involvement	–	–	
Pct-serum (median) (pg/mL)	143,551 (1.779–1000.0)	137,8185 (0.0–1000.0)	*p*:0.858
Pct-serum (mean) (pg/mL)	173,2376	214,39,382	
Pct-saliva (median) (pg/mL)	96,846 (23.906–1000.0)	90,138 (11.484–229.525)	*p*:0.159
Pct-saliva(mean) (pg/mL)	132,40,083	89,6360	

Statistical analysis revealed no significant difference in serum and salivary Pct levels between the patient and control groups (*p* > 0.05). Furthermore, no significant correlation was observed between disease activity and serum and salivary Pct values (*p* > 0.05). However, the serum Pct level in patients with active oral ulcers was found to be significantly higher than in patients without active oral ulcers (*p*: 0.003).

## Discussion

Behçet’s disease can manifest in multiple organs and systems, including the skin, eyes, joints, lungs, vessels, central nervous system, and gastrointestinal system, giving rise to various organ-specific clinical manifestations. The disease typically follows a course of exacerbations and remissions, with its activity diminishing over time. 1, 5.

The human oral cavity harbors various types of major bacteria that engage in intricate interactions with each other and the host immune system, establishing a stable symbiotic microenvironment in a state of health. However, disruptions in microbial flora balance contribute to the onset of both oral and systemic diseases. Conditions associated with this microbial imbalance encompass oral leukoplakia, oral lichen planus, systemic lupus erythematosus, periodontitis, peri-implantitis, oral cancers, obesity, HIV infection, inflammatory bowel disease, polycystic ovary syndrome, adverse pregnancy outcomes, pancreatic cancer, diabetes, rheumatoid arthritis, Alzheimer’s disease, and atherosclerosis. 3, 6. Although the pathogenesis of BD remains incompletely understood, it is hypothesized that infections, particularly involving Streptococcus sanguinis, and/or environmental factors may act as triggers for the symptomatology of the disease in individuals with a genetic predisposition 3, 7. In those genetically predisposed, chronic infection of oral mucosal structures, along with bacterial products, heat shock proteins (HSP), acute phase reactants, and other inflammatory mediators, stimulates a persistent systemic inflammatory response. Given that the disease typically initiates from oral mucosal surfaces, the local immune response to salivary microorganisms enters the systemic circulation. Consequently, it interacts with endothelial tissues, thereby elucidating the pathogenesis of the systemic symptoms observed in BD 2.

The pathogenesis of BD is intricately connected to genetic factors, with delayed-type hypersensitivity reactions to oral streptococci mediated by the IL-12 cytokine family identified as one of the initiating factors. Specifically, the four peptides of heat shock protein 65 (HSP-65) produced by Streptococcus sanguinis exhibit a 60–80% homology to human HSP-60 8. This resemblance leads to cross-reactivity between microbial HSPs and host antigens. Streptococcal antigens have been observed to stimulate the production of interleukin IL-6 and interferon-gamma (IFN-gamma) by peripheral T-cells in patients with BD 7. Additionally, there is an elevation in the spontaneous secretion of tumor necrosis factor-alpha (TNF-α), interleukin-6 (IL-6), and interleukin-8 (IL-8) in monocyte cultures obtained from BD patients. The increased levels of IL-8 may prove useful for the early detection of vascular involvement and assessment of BD activity 9.

Procalcitonin is a 116 amino acid peptide, serving as the precursor to calcitonin. Typically secreted by thyroid C cells for conversion into mature calcitonin to maintain homeostasis 10. In healthy individuals, serum Pct concentrations are not detectable. Pct is primarily released by peripheral blood mononuclear cells, and its levels are regulated by bacterial lipopolysaccharides and sepsis-associated cytokines.

In the context of severe bacterial infection and sepsis, Pct concentrations exhibit an increase starting around 4 h, reaching peak levels between 8 and 24 h 11, 12. Pct levels are higher in streptococcal infections compared to other microorganism infections 4. Notably, Pct levels are higher in streptococcal infections compared to infections caused by other microorganisms. Bellmann-Weiler et al. conducted a retrospective analysis of records from 61 hospitalized patients with community-acquired pneumonia (CAP) attributed to either Streptococcus pneumoniae or Legionella pneumophila. Their findings indicated that serum Pct levels were significantly lower in patients infected with L. pneumophila compared to those infected with S. pneumoniae 13.

According to the findings of this study, no statistically significant difference was observed in serum and salivary Pct levels between the patient and control groups (*p* > 0.05). However, it is crucial to note that infectious agents cannot be definitively excluded from the etiology. A study published in 2004 similarly reported no significant difference in serum Pct levels between active BD and the control group 14. The observation that serum Pct levels did not exhibit a correlation with disease activity scores, as seen in both Adam and Calıkoglu’s study and the current study, suggests limitations in the utilization of Pct as a marker for disease severity. Nevertheless, it is noteworthy that serum Pct levels were significantly higher in patients experiencing active oral aphthous ulcers (*p*: 0.003) compared to those without such ulcers. As previously discussed, microorganisms found in the saliva of BD patients are recognized to elevate serum Pct levels in various other diseases they contribute to 4, 13. Therefore, the elevated Pct levels during aphthous ulcer attacks underscore the significance of these microorganisms, implicated in the pathogenesis of BD, in the disease’s etiology.

This study reinforces the significance of infectious agents in the pathogenesis of BD. Pct emerges as a potentially important marker for monitoring oral aphthous ulcer attacks in the patient population. The finding that serum Pct values may be elevated in the presence of oral ulcers highlights its potential as a diagnostic indicator; however, further extensive and large-scale studies are necessary to validate its application in prognostic evaluations.

The limitations of this study include a restricted patient population, the absence of an untreated patient group, and the inability to assess patients both before and after treatment. Addressing these limitations in future research endeavors would contribute to a more comprehensive understanding of the role of Pct in the context of BD and its potential clinical applications.

## Data Availability

The data that support the fndings of this study are available from the corresponding author upon reasonable request.
